# A MR-PheWAS and bidirectional Mendelian randomization study: Exploring for causal relationships of pancreatic cancer

**DOI:** 10.1097/MD.0000000000040047

**Published:** 2024-10-11

**Authors:** Aiyu Guan, Zeming Li, Xingren Guo

**Affiliations:** a Department of Hepatobiliary Surgery, Yuncheng Central Hospital Affiliated to Shanxi Medical University, Yuncheng, Shanxi Province, China; b Department of Vascular Surgery, Yuncheng Central Hospital Affiliated to Shanxi Medical University, Yuncheng, Shanxi Province, China.

**Keywords:** Atlas, eosinophils, MR-pheWAS, pancreatic cancer, UK-biobank

## Abstract

It is unknown what causes pancreatic cancer. We conducted a phenome-wide Mendelian randomization analysis (MR-pheWAS), a bidirectional Mendelian study, and a systematic review of research in order to thoroughly investigate any causal association between pancreatic cancer and Atlas. We used phenome-wide Mendelian randomization analysis to test for associations between pancreatic cancer and 776 phenotypes (n = 452,264) of Atlas in the UK Biobank. Causality is confirmed by two-sample Mendelian randomization (MR) analysis using correlation found by false discovery rate correction. Simultaneously, a comprehensive evaluation of pancreatic cancer MR studies was conducted in order to complement our findings and harmonize the existing evidence. According to the inverse-variance-weighted model, a total of 41 out of 776 phenotypes had a nominal significance level (*P* < .05) genetic prediction association with pancreatic cancer. Only genetically predicted pancreatic cancer was shown to be linked with elevated eosinophil counts following false discovery rate correction (*P* = .031) when several tests were taken into account. Pancreatic cancer and eosinophils were shown to be positively causally associated to one another, establishing a self-loop, according to two-sample MR validation in the IEU database (OR = 1.011, 95% CI: 1.002–1.020, *P* = .010) (OR = 1.229, 95% CI: 1.037–1.458, *P* = .017). Although MR-pheWAS found a strong causal relationship between eosinophils and pancreatic cancer, it also found a negative exclusion value for each phenotype and a significant number of suggestive association phenotypes that offered guidance for further research.

## 1. Introduction

Although the prevalence of pancreatic cancer in malignant tumors is not great, during the past few decades, it has been the primary cause of mortality from cancer.^[1]^ Pancreatic cancer incidence is expected to double annually in the coming decades due to factors such as the changing global population’s age distribution, increased life expectancy, advancements in diagnostic technology, and an increase in patient risk factors such as high body mass index (BMI), diabetes, and smoking.^[2,3]^ The early symptoms are unusual, and the onset is concealed. When they visit a doctor, the majority of patients are in the middle or late stages and have missed the opportunity for radical surgery. Pancreatic cancer has become a hot topic in several studies due to its high mortality, short survival rate, and rising yearly incidence, which have resulted in a major health and financial burden.^[4]^

Conventional observational studies reveal that genetic variables are also significant in the development of pancreatic cancer.^[5]^ Genome-wide association studies (GWAS), which can also help guide patients’ treatment decisions and encourage high-risk families to conduct early screening, have further strengthened our understanding of the etiological mechanism of pancreatic cancer with the advancement of genome technology and the application of meta-analysis. Because genetic variants are randomly distributed at conception, Mendelian randomization (MR) is a causal correlation analysis approach that employs genetic variation as a tool variable to analyze exposure to illnesses. There has been a dramatic rise in the quantity of MR studies in recent years. Due to the small sample sizes and limited genetic variety employed in early MR research, the efficacy of these investigations is often low. However, the advancement of MR research has been aided by the identification of numerous genetic variations that are closely linked to particular biological traits, as well as the release of hundreds of thousands of summary data from GWAS regarding the connections between genetic variations, diseases, and exposure. Using MR, prior research has shown a causal association between pancreatic cancer, cholelithiasis, Crohn disease, type 2 diabetes mellitus, and fasting insulin levels.^[6–8]^ All of these MR investigations examine the causal association between exposure and pancreatic cancer, but their breadth of interest is limited since they are predicated on the theory of the disease’s origin. In order to uncover previously undiscovered characteristics, a hypothesis-free technique for determining causal relationships—known as phenome-wide Mendelian randomization analysis (MR-PheWAS)—has recently been developed.

**Figure 1.**
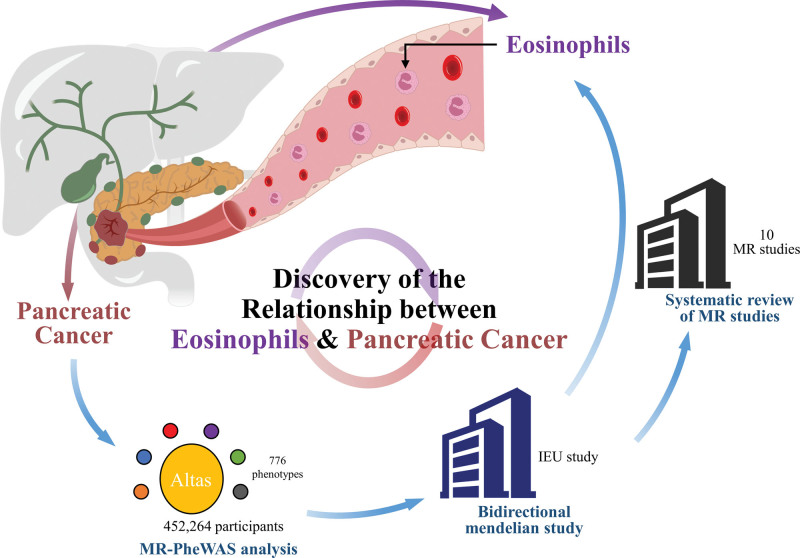
Study design overview. IEU = IEU open GWAS project, MR-PheWAS = phenome-wide Mendelian randomization analysis.

To comprehensively investigate the cause of pancreatic cancer, we used an MR-PheWAS to identify a novel causal link that was missed by earlier research (Fig. 1). Our analysis focused on 776 phenotypes in UK Biobank, which were represented by 9,113,133 genetic variants.^[9]^ Additionally, we summarized the pancreatic cancer GWAS genetic data, which comprised 9040 cases and 12,496 control participants.^[10]^

## 2. Methods

### 2.1. An Atlas of genetic associations in UK Biobank

In the UK Biobank Gene Association Atlas, MR-pheWAS was conducted. Five lakhs three thousand three hundred twenty-five people (273,453 females) between the ages of 40 and 69 who were recruited from 22 assessment centers in England, Scotland, and Wales between 2006 and 2010 made up the UK Biobank cohort.^[11,12]^ Affymetrix UK BiLEVE Axiom array was used for the first 50,000 individuals’ genotyping, whereas Affymetrix UK Biobank Axiom array was used for the remaining 450,000 people. Interpolation and quality checking were handled by the Wellcome Trust Centre for Human Genetics.^[13]^ With the use of an openly accessible database, we created the biggest genetic association map to date for the 116 nonbinary and 660 binary variables for 452,264 UK Biobank White individuals in the atlas we presented (Table S1, Supplemental Digital Content, http://links.lww.com/MD/N718). In writing, each participant provided informed permission.

### 2.2. Identifying the genetic instruments for pancreatic cancer

From the most recent GWAS, we were able to identify genetic markers for pancreatic cancer.^[10]^ Under the conventional GWAS criterion (*P*-value < 5 × 10^‐8^) our meta-analysis found 21 single nucleotide polymorphisms (SNPs) (Table S2, Supplemental Digital Content, http://links.lww.com/MD/N718). After applying LDlink (R2 ≤ 0.001) for linkage disequilibrium trimming^[14]^, the MR-pheWAS analysis revealed a total of 17 independent associated variants, comprising 5 novel variants that were successfully replicated in this publication and 12 SNPs that had previously been found by GWAS in pancreatic cancer.

### 2.3. Phenome-wide Mendelian randomization analysis

We utilized PheWAS and the MR approach to examine the relationships between pancreatic cancer and other phenotypes. To evaluate these relationships in the current work, we employed inverse variance weighting, or IVW. *P* values <.05 following false discovery rate correction were deemed significant due to the possible connection between several phenotypes.^[15]^ We employed MR Egger test to identify possible multidirectional effects and IVW and MR Egger regression to identify heterogeneity for these significant relationships. R 4.1.3’s TwosampleMR package, version 0.5.6, was used for this investigation.

### 2.4. Replication analysis and reverse analysis of MR-pheWAS findings

Using the GWAS summary data in IEU, we further validated the favorable results of MR-pheWAS using a two-sample MR analysis. Second, two-sample MR was carried out to confirm the existence of reverse causality. The causative association between pancreatic cancer and eosinophil count was evaluated, and sensitivity analysis was carried out to examine the reliability of the results.

### 2.5. Systematic review of MR studies on pancreatic cancer

Since there aren’t many MR studies on pancreatic cancer at the moment and our study only produced one positive result, there is still a significant knowledge gap about the disease. Because MR studies are labor-intensive and time-consuming to conduct, they have a special value in examining causality. To supplement the correlations found in our study, we also thoroughly reviewed the pancreatic cancer MR database. We used the following search technique to find publications up to February 3, 2024: “Mendelian randomization analysis” and “pancreatic cancer.” We retrieved information on the study’s outcomes, the initial author, the year it was published, the genetic techniques employed, and the quantity of cases and controls.

## 3. Results

### 3.1. MR-PheWAS analysis

**Figure 2.**
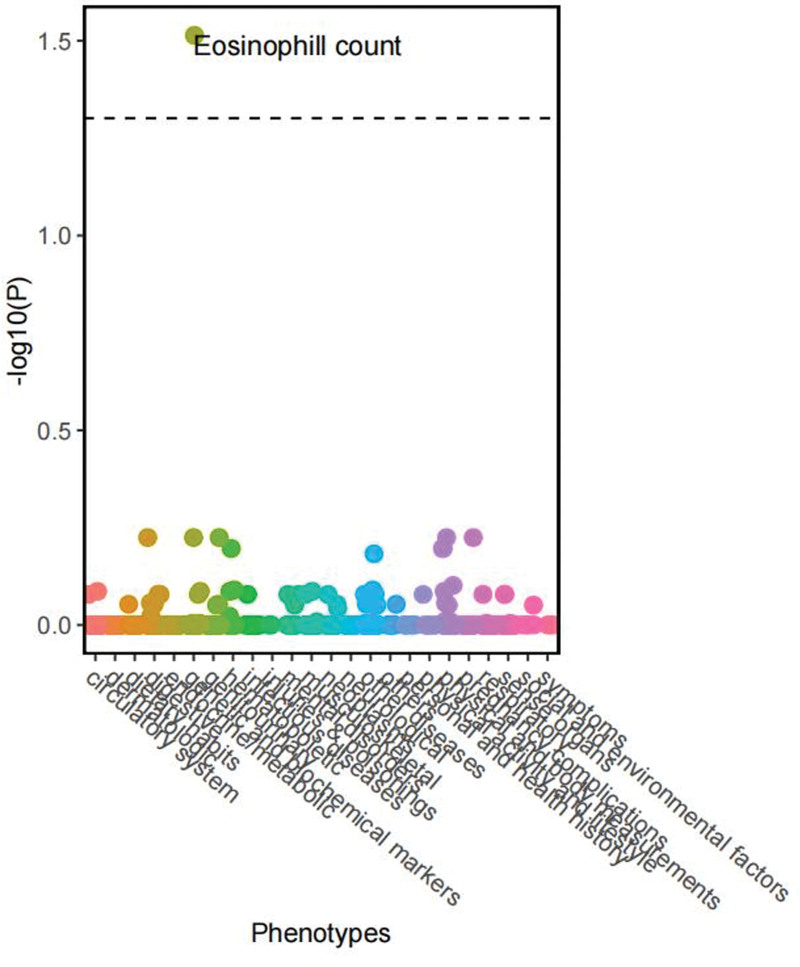
MR-pheWAS plot showing the overall distribution of findings according to categories. The dotted line reflects the *P* value after correction of FDR (0.05). FDR = false discovery rate, MR-PheWAS = phenome-wide Mendelian randomization analysis.

MR-PheWAS analysis was performed using 452,264 white pedigrees from the UK Biobank Atlas. 776 qualities total—116 non-binary and 660 binary—were included in the Atlas. Hospital episode statistics (i.e., information gathered during hospital admissions), self-reported characteristics at enrollment (e.g., self-reported depression), baseline measures (e.g., height), and cancer diagnoses from relevant UK cancer registries were among these phenotypes. A total of 41 phenotypes were found to be nominally associated with pancreatic cancer at the genetic predictive level (*P* < .05), and sensitivity analyses were conducted (Tables S3, S4, and S5, Supplemental Digital Content, http://links.lww.com/MD/N718). After false discovery rate correction accounting for multiple testing, elevated eosinophil levels were linked to genetically predicted pancreatic cancer (Fig. 2).

### 3.2. Bidirectional Mendelian randomization

**Figure 3.**
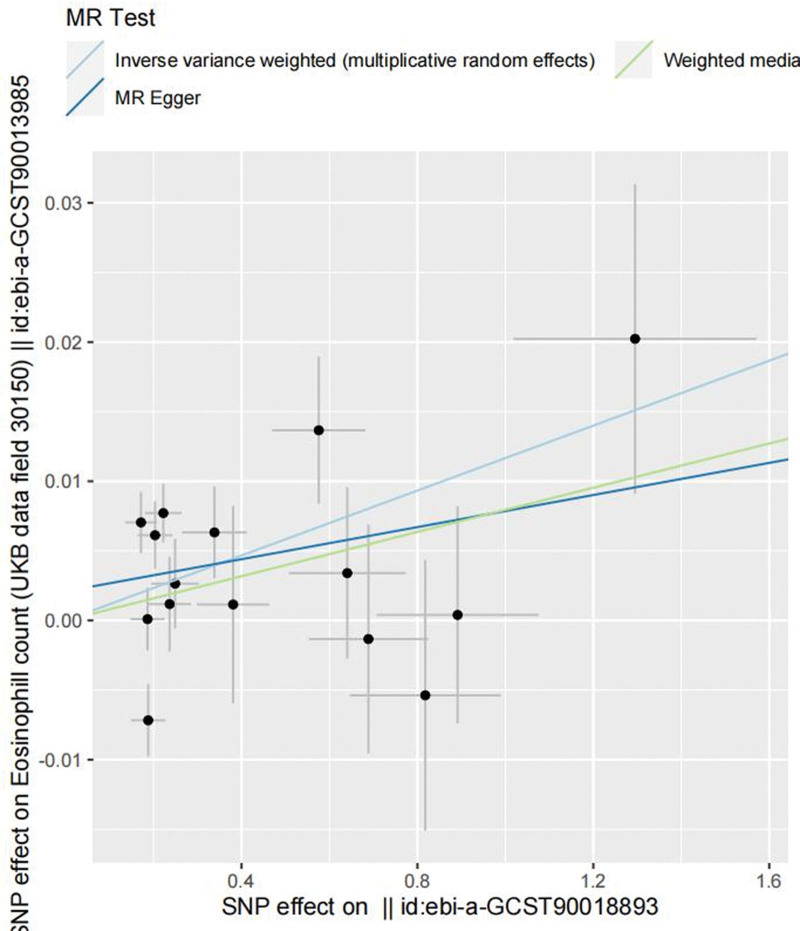
The horizontal coordinate is the effect of a single SNP on pancreatic cancer, and the vertical coordinate is the effect of a single SNP on eosinophils, with eosinophil counts rising as the risk of pancreatic cancer increased. SNP = single nucleotide polymorphisms.

**Figure 4.**
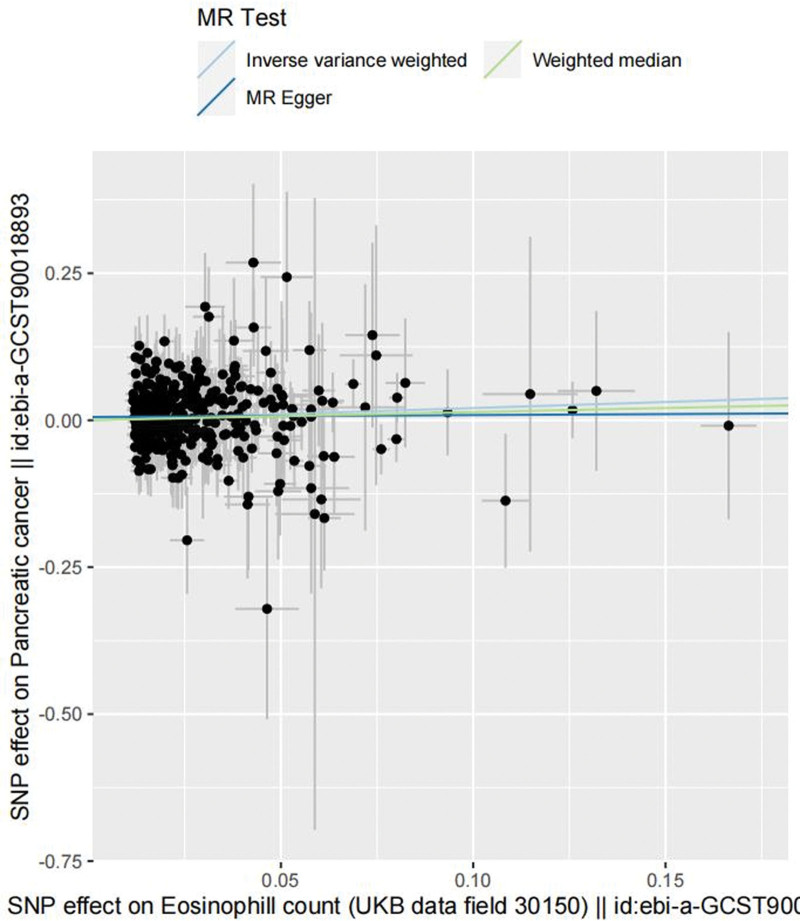
The horizontal coordinate is the effect of a single SNP on eosinophils, and the vertical coordinate is the effect of a single SNP on pancreatic cancer; as eosinophil counts increased, so did the risk of pancreatic cancer. SNP = single nucleotide polymorphisms.

We used data from the IEU open gwas project (https://gwas.mrcieu.ac.uk/), where the research data for pancreatic cancer comprised 1196 pancreatic cancer patients and 475,049 controls, for additional validation of MR-pheWAS positive results. Three lakhs ninety-five thousand nine hundred forty-nine samples were included in the eosinophil count (Table 1). In this work, inverse-variance weighted (IVW) was primarily used for two-sample Mendelian randomization analysis. For no heterogeneity, a fixed-effects model was used, and for heterogeneity, a random-effects model was used. The weighted median technique and MR-Egger regression were used to complement the IVW findings. Since polytropy and other factors might have an impact on traditional IVW analysis techniques, this study used sensitivity analysis to evaluate the validity and robustness of IVW conclusions. *P* values for the SNPs were <5 × 10^‐6^ when pancreatic cancer was used as the exposure. In the end, 15 SNPs were found to be suitable instrumental variables (IVs) after meeting the 3 major assumptions of Mendelian randomization (Table S6, Supplemental Digital Content, http://links.lww.com/MD/N718). The Cochran Q test for the test of heterogeneity IVW (*P* = .001) and MR-Egger regression (*P* = .001) revealed heterogeneity in the SNPs (Table S7, Supplemental Digital Content, http://links.lww.com/MD/N718). Indicating that the random effects model of IVW was used for the analysis. The results of the IVW analysis of the random effects model indicated a significant positive correlation (OR = 1.011, 95% CI: 1.002–1.020, *P* = .010) between pancreatic cancer and eosinophil counts; the results of the weighted median (OR = 1.007, 95% CI: 1.008–1.017, *P* = .079) and MR-Egger regression (OR = 1.005, 95% CI: 1.006–1.023, *P* = .524) indicated a significant but non-statistically significant correlation between the 2, as well as the direction of the causal effect obtained by the 3 methods was identical (Table 2 and Fig. 3). Since the MR-Egger test intercept value and 0 did not vary statistically (*P* = .45) (Fig. S1, Supplemental Digital Content, http://links.lww.com/MD/N717 and Table S7, Supplemental Digital Content, http://links.lww.com/MD/N717), we may conclude that SNPs do not exhibit horizontal pleiotropy. The *P*-value of SNPs was <5 × 10^‐8^ when eosinophil count was used as an exposure. Ultimately, 357 SNPs were selected as appropriate IVs (Table S8, Supplemental Digital Content, http://links.lww.com/MD/N718). The IVW analysis of fixed-effects modeling also revealed a significant positive correlation between pancreatic cancer and eosinophil count (OR = 1.229, 95% CI: 1.037–1.458, *P* = .017); the weighted median results (OR = 1.007, 95% CI: 0.853–1.545, *P* = .361) and the MR-Egger regression (OR = 1.005, 95% CI: 0.735–1.452, *P* = .849) also revealed a significant positive correlation between the 2, but it was not statistically significant (Table 3 and Fig. 4). The heterogeneity test indicated that there was no heterogeneity in the SNPs, and the MR-Egger test revealed no statistically significant difference between the Egger-intercept value and 0 (*P* = .45) (Fig. S2, Supplemental Digital Content, http://links.lww.com/MD/N717 and Table S9, Supplemental Digital Content, http://links.lww.com/MD/N718). In the bidirectional Mendelian randomization study, the IVs’ *F*-values were all >10 (Tables S10 and S11, Supplemental Digital Content, http://links.lww.com/MD/N718). In the leave-one-out analysis, the sequential removal of each SNP did not reveal any SNPs with a substantial impact on the estimates of the causal association (Figs. S3–S6, Supplemental Digital Content, http://links.lww.com/MD/N717).

**Table 1:** GWAS database studies data information.

Phenotype	ID	Sample size	Number of SNPs	Population	Year
Eosinophil count	ebi-a-GCST90013985	395,946	10,783,686	European	2021
Pancreatic cancer	ebi-a-GCST90018893	476,245	24,195,229	European	2021

**Table 2:** Main analytical methods of MR (exposure: pancreatic cancer outcome: eosinophil count).

	Id. exposure	Id. outcome	Outcome	Exposure	Method	NSNP	*b*	SE	*P*-val	lo_ci	up_ci	or	or_lci95	or_uci95
1	ebi-a-GCST90018893	ebi-a-GCST90013985	Eosinophil count (UKB data field 30,150) ‖ id:ebi-a-GCST90013985	‖ id:ebi-a-GCST90018893	Inverse variance weighted (multiplicative random effects)	15	0.011663435	0.00454731	.010320304	0.002750706	0.020576163	1.011731718	1.002754493	1.020789312
2	ebi-a-GCST90018893	ebi-a-GCST90013985	Eosinophil count (UKB data field 30,150) ‖ id:ebi-a-GCST90013985	‖ id:ebi-a-GCST90018893	MR Egger	15	0.005765471	0.008821907	.524799767	‐0.011525468	0.023056409	1.005782123	0.988540696	1.023324263
3	ebi-a-GCST90018893	ebi-a-GCST90013985	Eosinophil count (UKB data field 30,150) ‖ id:ebi-a-GCST90013985	‖ id:ebi-a-GCST90018893	Weighted median	15	0.007945405	0.004516525	.078546031	‐0.000906984	0.016797794	1.007977053	0.999093427	1.01693967

b = beta, MR = Mendelian randomization, SE = standard error.

**Table 3:** Main analytical methods of MR (exposure: eosinophil count outcome: pancreatic cancer).

	Id. exposure	Id. outcome	Outcome	Exposure	Method	NSNP	*b*	SE	*P*-val	lo_ci	up_ci	or	or_lci95	or_uci95
1	ebi-a-GCST90013985	ebi-a-GCST90018893	Pancreatic cancer ‖ id:ebi-a-GCST90018893	Eosinophil count (UKB data field 30,150) ‖ id:ebi-a-GCST90013985	Inverse variance weighted	356	0.204857614	0.086935309	.018451177	0.034464409	0.375250819	1.227350295	1.035065189	1.4553564
2	ebi-a-GCST90013985	ebi-a-GCST90018893	Pancreatic cancer ‖ id:ebi-a-GCST90018893	Eosinophil count (UKB data field 30,150) ‖ id:ebi-a-GCST90013985	MR Egger	356	0.036609624	0.173718785	.833210841	-0.303879194	0.377098442	1.037288009	0.73795001	1.458047835
3	ebi-a-GCST90013985	ebi-a-GCST90018893	Pancreatic cancer ‖ id:ebi-a-GCST90018893	Eosinophil count (UKB data field 30,150) ‖ id:ebi-a-GCST90013985	Weighted median	356	0.138298647	0.144193288	.337498365	-0.144320198	0.420917492	1.148318441	0.865610537	1.523358584

MR = Mendelian randomization.

### 3.3. Systematic review of MR studies on pancreatic cancer

Ten pertinent papers out of the 88 that were obtained from the PubMed database were included in the review. The analysis showed a correlation between an elevated risk of pancreatic cancer and genetically predicted pancreatic fat, high-density lipoprotein, BMI particles, BMI, fasting insulin level, Crohn illness, intestinal flora, and C-reactive protein. In contrast, retinol, IL-1RA, and genetically predicted very low-density lipoprotein particles were linked to a decreased risk of pancreatic cancer (Table 4).

**Table 4:** Associations of higher genetically proxied exposures with pancreatic cancer from a systematic review of Mendelian randomization studies. Estimates were obtained from the inverse-variance weighted method.

First author	Exposure	Outcome	Cases	Controls	OR (95%CI)	*P*
Maina JG	BMI	Pancreatic cancer	457,270		1.073 [1.018–1.130]	.0037
li ZQ	CRP	Pancreatic cancer	204,402		1.441 [1.064–1.950]	2.71E‐39
Yarmolinsky J	interleukin-23 receptor concentrations	Pancreatic cancer	59,969		1.42 [1.200–1.690]	*q* = 0.055
Fan R	Chronic Constipation	Pancreatic cancer	36,022	341,255	2.29 [1.422–3.690]	.001
Jiang ZC	Ruminococcaceae (UCG011)	Pancreatic cancer	18,340		1.433 [1.072–1.916]	.015
Jiang ZC	Streptococcus	Pancreatic cancer	18,340		1.712 [1.071–1.736]	.025
Rao M	Cholelithiasis	Pancreatic cancer	19,023	195,144	1.676 [1.228–2.288]	.001
Min Y	Crohn disease	Pancreatic cancer	13,768	33,977	1.111 [1.015–1.213]	.022
Zhang XN	Retinol	Pancreatic cancer	1960		0.747 [0.584–0.955]	.020
Yuan S	Interleukin-1 receptor	Pancreatic cancer	21,758		0.860 [0.770–0.960]	.009
Brennan P	Fasting insulin	Pancreatic cancer	7110	7264	1.660 [1.050–2.630]	.03
Sun R	HDL	Pancreatic cancer	115,078		1.290 [1.050–1.590]	.017
Sun R	VLDL	Pancreatic cancer	115,078		0.740 [0.550–0.990]	.043
Yamazaki H	Pancreas fat	Pancreatic cancer	25,617		2.460 [1.380–4.400]	.002

BMI = body mass index, CI = confidence interval, CRP = C-reactive protein, HDL = high-density lipoprotein, OR = odds ratio, VLDL = very low-density lipoprotein.

## 4. Discussion

With a high death rate and a low incidence of surgery following diagnosis, pancreatic cancer is and will continue to be one of the unmet requirements of cancer. The etiologic foundation of pancreatic cancer remains poorly understood after 2 decades of intensive research, which hinders the development of strategies to lessen the disease’s impact. In contrast, recent studies on malignancies of the liver, lung, colon, breast, and thyroid have found risk factors for these diseases.^[16–19]^ In this investigation, MR-pheWAS confirmed the reciprocal causation in bidirectional MR and discovered compelling evidence for the correlation between eosinophils and pancreatic cancer. We carried out a comprehensive evaluation of MR studies with outcomes of pancreatic cancer, which significantly enhanced the evidence of our investigation, as MR-PheWAS is not capable of doing inverse analysis. Furthermore, we offer more thorough guidance for researching the basic processes and distinct pathways involved in pancreatic cancer.

The current MR analysis, an epidemiological study based on hypothesis,^[20]^ is insufficient for our investigation of multiple phenotypes, despite the abundance of GWAS data offering SNPs that can be used as IVs for MR analysis, which has the advantage of avoiding many of the limitations of traditional observational studies. As a result, the combination of MR and PheWAS enables us to test the relationship between multiple traits and pancreatic cancer risk in a hypothesis-free manner.^[21]^ In order to reduce the amount of human resources needed and the number of associations that do not need to be explored in order to provide a sample for the study, we can down-prioritize these phenotypes in future studies thanks to the more negative exclusion values and positive endpoints that the current study’s exploration of causal associations offers.

White blood cells called eosinophils are produced from pluripotent hematopoietic stem cells found in bone marrow.^[22]^ They are mostly found in the intestinal mucosa, where they maintain innate immunity and carry out effects and immunomodulatory functions. They are involved in several forms of immunity connected to innate immunity and adaptive immunity in the human body.^[23]^ Paul Ehrlich made the initial observation of eosinophils in 1879 and noted that helminth and asthmatic infections had greater eosinophil counts.^[24]^ This finding showed that eosinophils were advantageous to the host’s defense against parasite infection. Apart from their direct interaction with other cells, eosinophils control the development of inflammation and immunity through the synthesis, storage, and release of cytokines.^[25,26]^ As far back as 120 years ago, there was a report on the peripheral eosinophil count in cancer patients, despite the fact that the majority of the early research on eosinophils was on their utility in allergic disorders and parasitic infections.^[27]^ The “unpopular” discovery was the result of less thorough eosinophil research in the past. But as science continues to advance, our knowledge of eosinophils is progressively shifting. Although the presence and prognosis of tumor infiltrating eosinophils and/or peripheral blood eosinophils have been documented,^[28,29]^ eosinophils have been shown as a component of the tumor microenvironment to impact tumor growth.^[30]^ By expressing cytotoxic markers as natural killer group 2 member D, eosinophils cause cytotoxicity to liver cancer tumor cells.^[31]^ Conversely, eosinophil infiltration is linked to a poor prognosis for other tumor forms, such as cervical cancer, oral squamous cell carcinoma, and Hodgkin lymphoma.^[32,33]^ As a result, there is ongoing debate over eosinophils’ function in tumor microenvironment. Despite reports of eosinophils in pancreatic cancer, nothing is known regarding their involvement in the disease.^[34–36]^

Studies have indicated that eosinophils and pancreatic cancer may be associated with inflammatory response, IL-5, IL-18, and degranulation, even if the precise mechanism behind this association is still unclear. Research has demonstrated that the pathological characteristic of pancreatic cancer is pancreatic fibrosis, and that IL-5 contributes to the development of pancreatic fibrosis. After injection, animals without the IL-5 gene showed less eosinophil infiltration and collagen production than mice with the IL-5 gene.^[37]^ Furthermore, inflammatory factors have a role in carcinogenesis, carcinoma invasion, and metastasis, among other phases of the cancer formation process.^[38]^ Research has revealed that cyanin injection causes mice to exhibit worsened chronic pancreatitis and eosinophil infiltration, which speeds up the growth of pancreatic tumors.^[39]^ Periostin has been shown to enhance eosinophil migration and activation, which may be the mechanism behind the elevation of eosinophil counts in chronic pancreatitis.^[40,41]^ Additionally, it was shown that soluble IL-5 boosted the expression of IL-5Rα in pancreatic ductal adenocarcinoma (PDAC) cells, facilitated PDAC cell motility and STAT5 activation, and that blocking this receptor in PDAC was linked to a reduction in tumor development and metastasis.^[42]^ Pathogenic eosinophils can be generated by IL-18 from bone marrow progenitor cells.^[43,44]^ By releasing peroxidase particles, eosinophils activate transforming growth factor-β and MUC2, hence promoting the growth of malignant tumors. Furthermore, one source of transforming growth factor-β is eosinophils.^[45]^ Murli et al discovered a connection between human pancreatic fibrosis and malignant tumors and the degranulation of mast cells and eosinophils.^[36]^ Additionally, our research revealed a direct association between pancreatic cancer and eosinophils, creating a vicious cycle that might explain why immune checkpoint inhibitors have a poor track record when it comes to treating pancreatic cancer by targeting eosinophils.

The benefit of MR-pheWAS is its ability to analyze the association between many phenotypes and pancreatic cancer risk by utilizing sizable GWAS data sets. Our present study does, however, nonetheless have a number of possible drawbacks. First of all, the only genetic tool variables available to us for studying phenotype are those that already exist. Second, hospital records are used to determine the majority of instances in the analysis; however, conditions that do not necessitate hospitalization are not covered. Third, there is a good chance that the numerous modifications made will result in misleading negative results. Fourthly, the European population is the only one on which our study is currently focused, and extrapolating to other races will require a large amount of cross-racial or other racial GWAS data. Lastly, we are still unable to establish the causal link between minor effects, even when we limit our analysis to the characteristic for which genetic techniques can account for at least 0.1% of phenotypic variance.

## 5. Conclusion

To put it succinctly, our systematic review and phenome-wide MR reveal the possible influence of pancreatic cancer on health-related traits. The findings of our study indicate that eosinophils negatively impact pancreatic cancer, and the eosinophil count may also be elevated by pancreatic cancer. Second, it has been established that none of the 775 types is the primary risk factor for pancreatic cancer, which makes it easier to avoid wasting time and resources on pointless study in the future; finally, for the suggestive association phenotype with substantial causal connection but not multiple corrections, it is conceivable to clarify a greater link with pancreatic cancer with the introduction of a bigger GWAS data set in the future, accompanied by a higher quantity of effects.

## Acknowledgments

We thank all the people who offered help with this study.

## Author contributions

**Conceptualization:** Aiyu Guan.

**Data curation:** Aiyu Guan, Xingren Guo.

**Formal analysis:** Xingren Guo.

**Investigation:** Zeming Li, Xingren Guo.

**Methodology:** Zeming Li, Xingren Guo.

**Project administration:** Aiyu Guan, Zeming Li.

**Resources:** Aiyu Guan.

**Supervision:** Aiyu Guan, Xingren Guo.

**Software:** Zeming Li.

**Validation:** Aiyu Guan.

**Visualization:** Aiyu Guan.

**Writing – original draft:** Aiyu Guan, Xingren Guo.

**Writing – review & editing:** Xingren Guo.

## Supplementary Material


