# Supplementary material for: A MR-PheWAS and bidirectional Mendelian randomization study: Exploring for causal relationships of pancreatic cancer
Source: Medicine (Baltimore). 2024 Oct 11;103(41):e40047. doi: 10.1097/MD.0000000000040047 (PMC11479532; doi:10.1097/MD.0000000000040047)
Supplement: Supplementary file 2 [file medi-103-e40047-s002.pdf]

Supplement Figure 1

Scatter plot of sensitivity analysis(exposure:Pancreatic cancer ;outcome:Eosinophill count)

Supplement Figure 2

Scatter plot of sensitivity analysis(exposure:Eosinophill count;outcome:Pancreatic cancer)

Supplement Figure 3

Leave-one-out analysis plots (exposure:Eosinophill count;outcome:Pancreatic cancer)

Supplement Figure 4

Forest map(exposure:Eosinophill count;outcome:Pancreatic cancer)

Supplement Figure 5

Leave-one-out analysis plots(exposure:Pancreatic cancer ;outcome:Eosinophill count)

Supplement Figure 6

Forest map(exposure:Pancreatic cancer ;outcome:Eosinophill count)

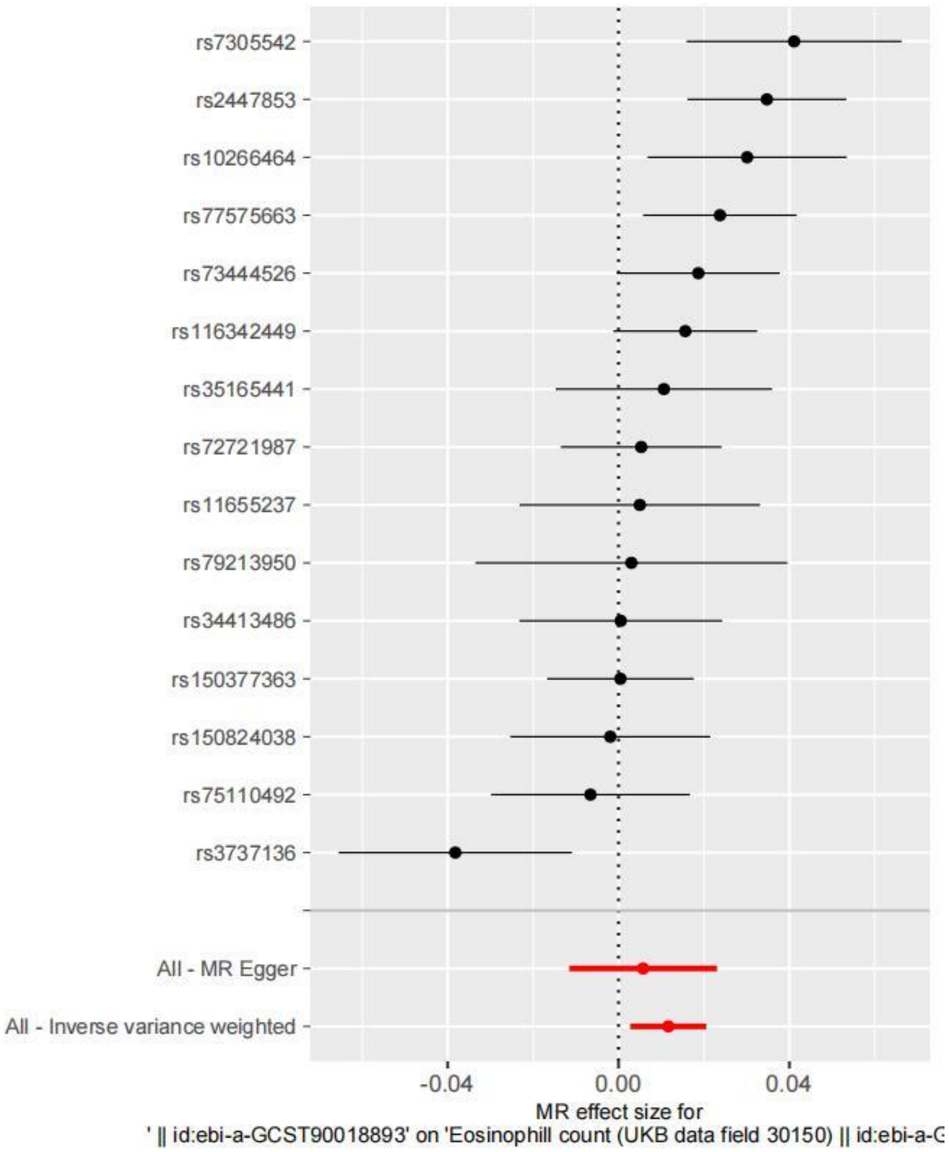
rs7305542
rs2447853
rs10266464
rs77575663
rs73444526
rs116342449
rs35165441
rs72721987
rs11655237
rs79213950
rs34413486
rs150377363
rs150824038
rs75110492
rs3737136
All - MR Egger
All - Inverse variance weighted
-0.04
0.00
0.04
MR effect size for
' || id:ebi-a-GCST90018893' on 'Eosinophill count (UKB data field 30150) || id:ebi-a-G

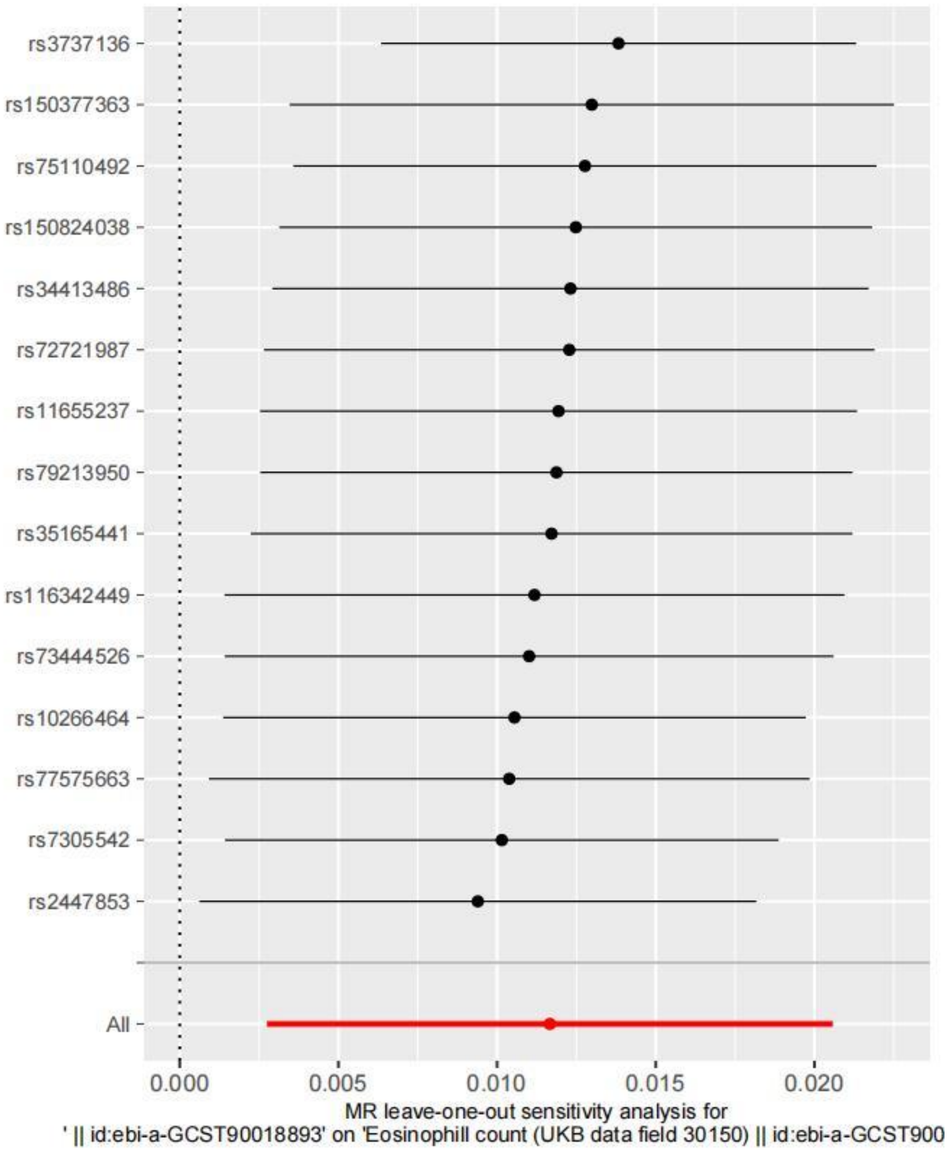
rs3737136
rs150377363
rs75110492
rs150824038
rs34413486
rs72721987
rs11655237
rs79213950
rs35165441
rs116342449
rs73444526
rs10266464
rs77575663
rs7305542
rs2447853
All
0.000
0.005
0.010
0.015
0.020
MR leave-one-out sensitivity analysis for
' || id:ebi-a-GCST90018893' on 'Eosinophill count (UKB data field 30150) || id:ebi-a-GCST900

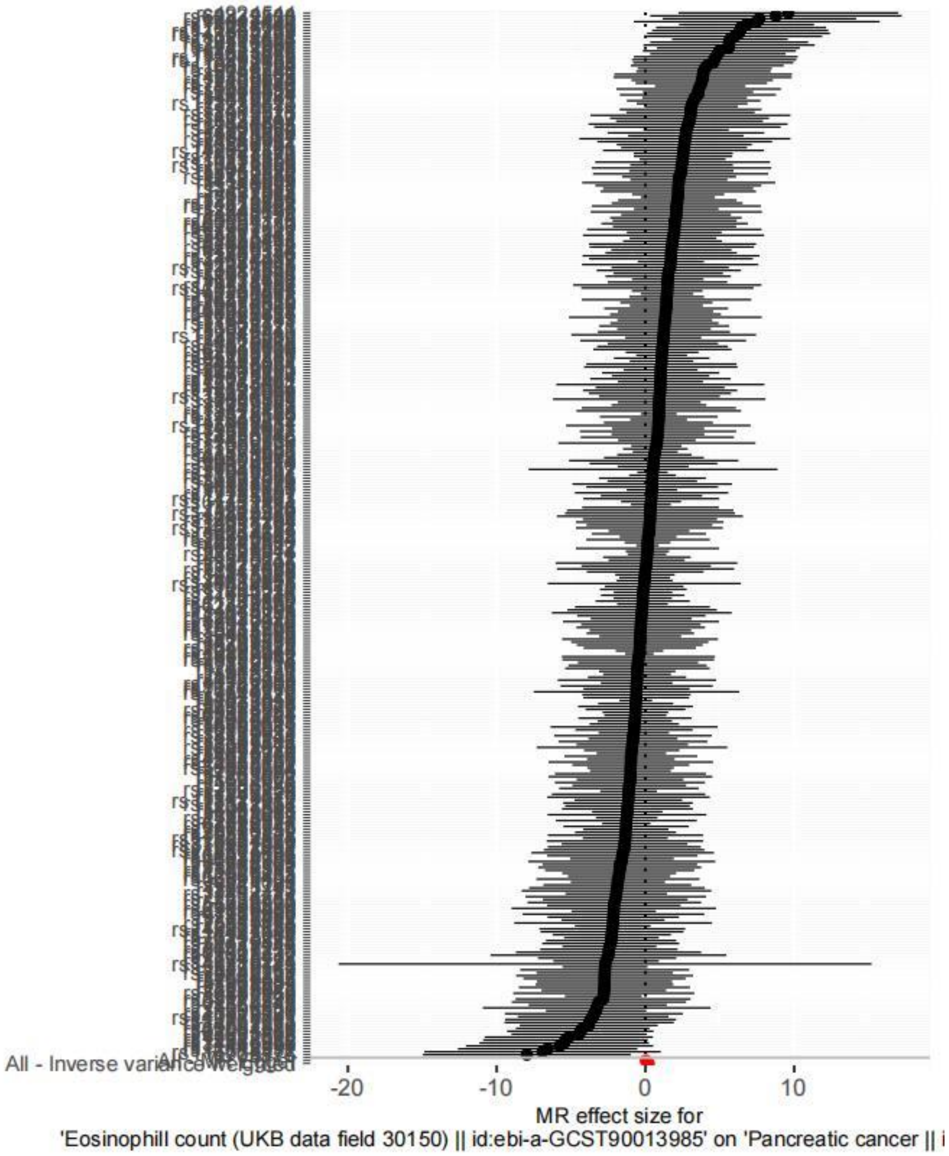
All - Inverse variance weighted
-20
-10
0
10
MR effect size for
'Eosinophill count (UKB data field 30150) || id:ebi-a-GCST90013985' on 'Pancreatic cancer || i

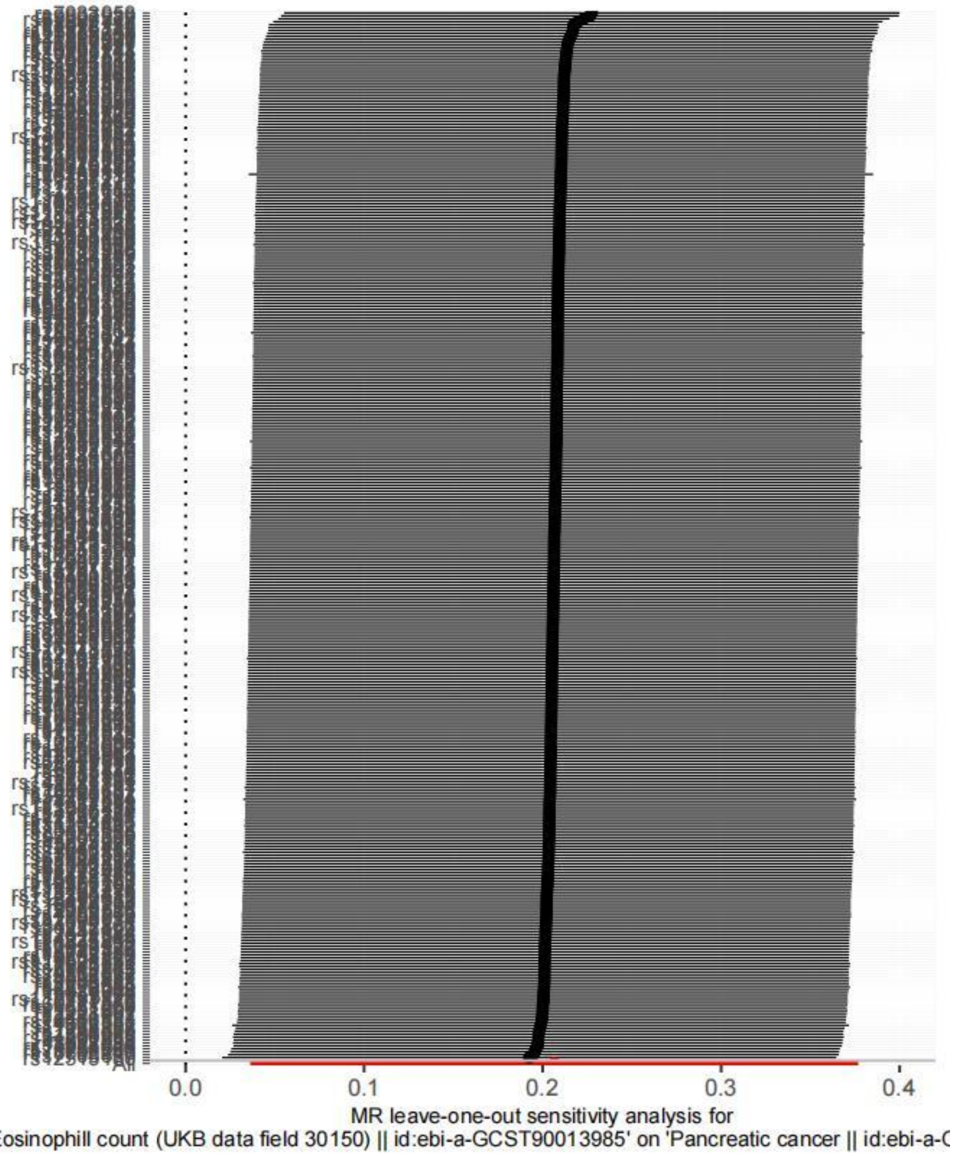
All
0.0
0.1
0.2
0.3
0.4
MR leave-one-out sensitivity analysis for
'Eosinophill count (UKB data field 30150) || id:ebi-a-GCST90013985' on 'Pancreatic cancer || id:ebi-a-[illegible]

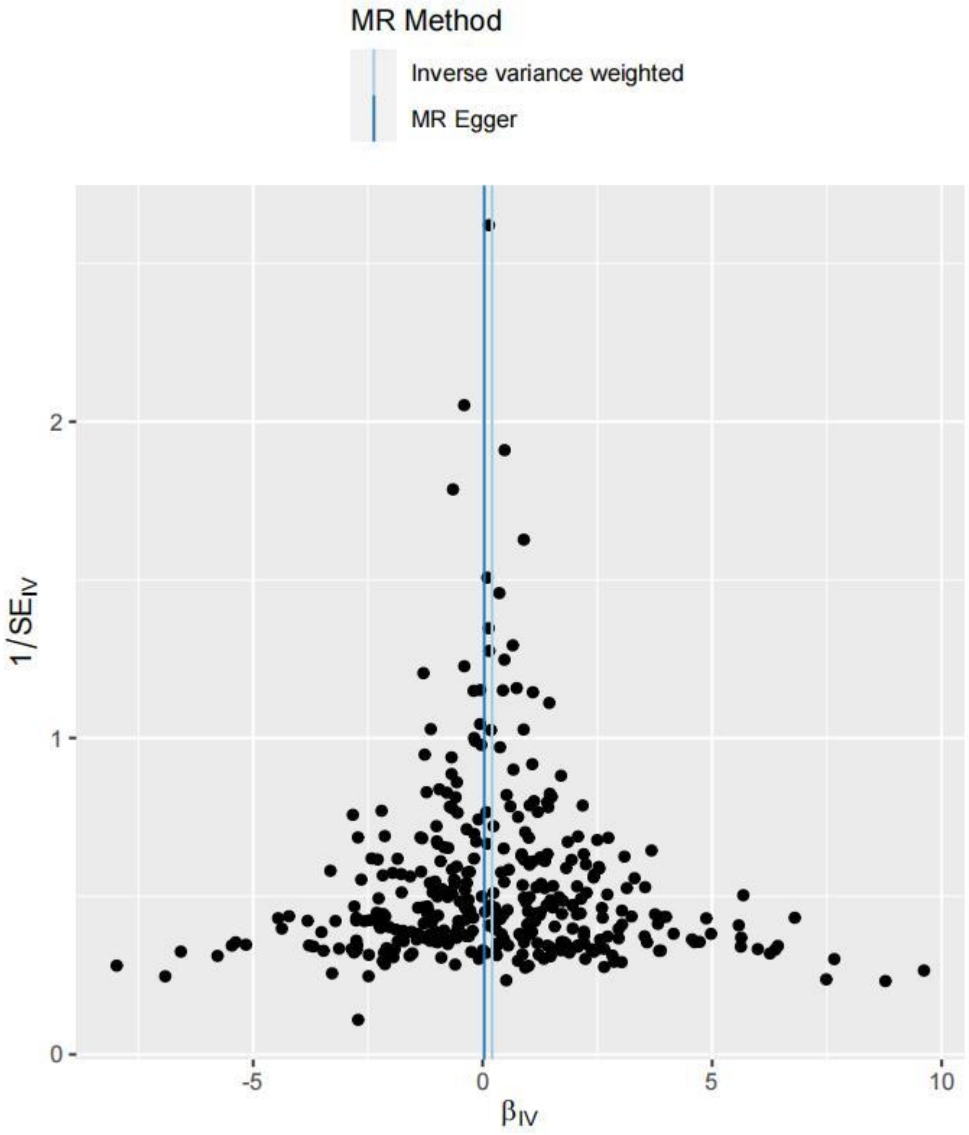
MR Method
Inverse variance weighted
MR Egger
1/SE$_{IV}$
2
1
0
-5
0
5
10
β$_{IV}$

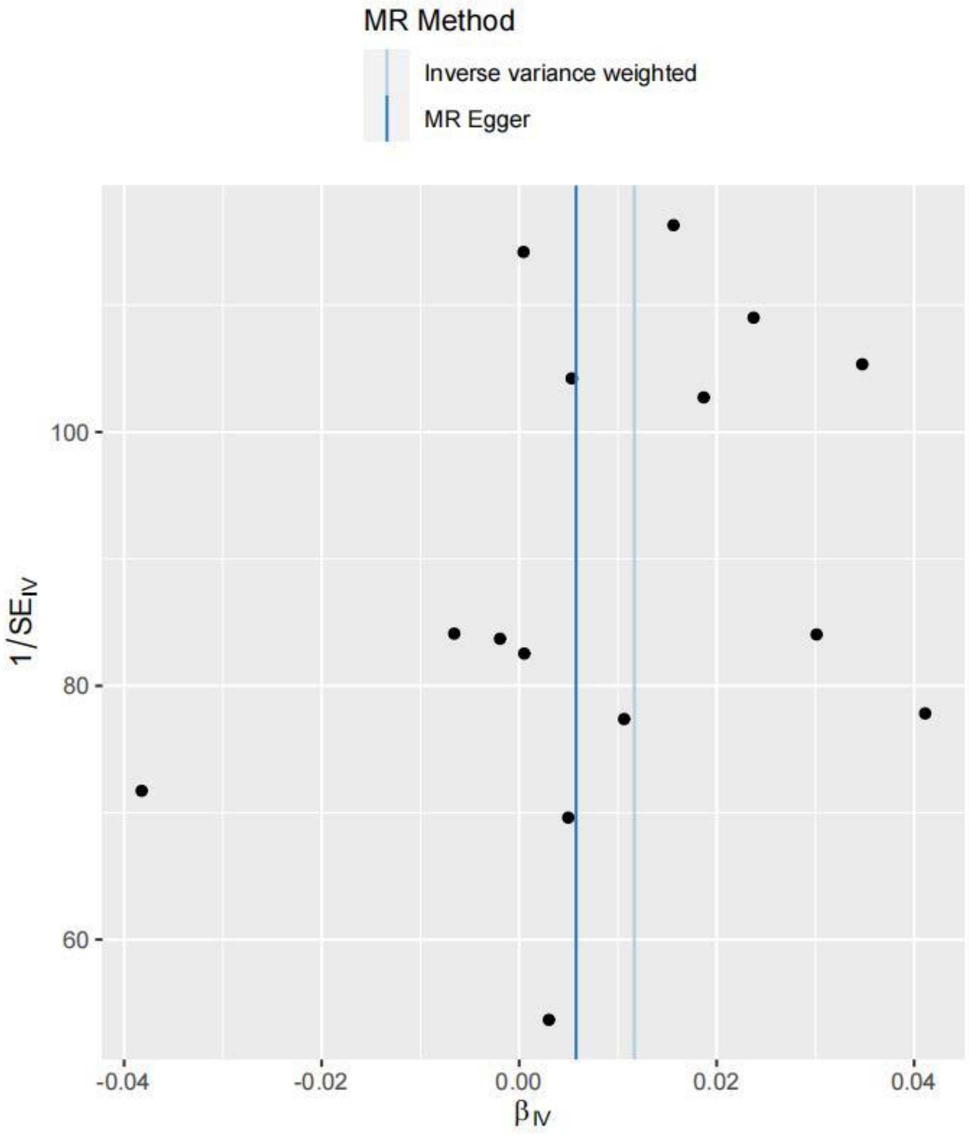
MR Method
Inverse variance weighted
MR Egger
100
80
60
1/SE_IV
-0.04
-0.02
0.00
0.02
0.04
β_IV
